# Somaclonal variation in Saccharum spp.: unraveling its potential despite current neglect

**DOI:** 10.18699/vjgb-26-42

**Published:** 2026-05

**Authors:** S. Munir, M.A.B. Jaffar, S. Yasmeen, M.T. Khan, I.A. Khan

**Affiliations:** Sugarcane Biotechnology Group, Nuclear Institute of Agriculture (NIA), Tando Jam, Pakistan Plant Breeding and Genetics Division, Nuclear Institute for Agriculture and Biology, Faisalabad, Pakistan; Sugarcane Biotechnology Group, Nuclear Institute of Agriculture (NIA), Tando Jam, Pakistan; Sugarcane Biotechnology Group, Nuclear Institute of Agriculture (NIA), Tando Jam, Pakistan; Agricultural Biotechnology Division, National Institute for Biotechnology and Genetic Engineering, Faisalabad, Pakistan; Sugarcane Biotechnology Group, Nuclear Institute of Agriculture (NIA), Tando Jam, Pakistan

**Keywords:** callus, genetics, in vitro culture, markers, regeneration, somaclonal variation, sugarcane, каллус, генетика, культивирование in vitro, маркеры, регенерация, сомаклональная изменчивость, сахарный тростник

## Abstract

Hybridization of different landraces or wild crop species facilitates genetic recombination and leads to the development of improved cultivars, particularly in sexually propagated crops. In contrast, genetic recombination via hybridization in asexually propagated crops like sugarcane (Saccharum spp. hybrid) is challenging due to self or cross incompatibility (low fertility). Such crops can be improved by somaclonal variation, which is achieved by tissue culture techniques. As a major contributor to global sugar and bioethanol production, sugarcane suffers substantial yield loss due to various biotic or abiotic stresses, which may be attributed to its poor resistance mechanism. Despite the potential of in vitro culture techniques, somaclonal variation remains underexplored in sugarcane breeding programs. To address the challenges posed to sugarcane under changing environmental dynamics, this review critically evaluates the role of somaclonal variation in sugarcane variety development, its underlying mechanism, practical applications, and factors affecting its occurrence. This review also discusses the limitations and challenges in the practical implementation of this technique in variety development, resulting in its neglect in modern breeding efforts. The focus on the potential of somaclonal variation, sustained by cutting-edge approaches, can unlock its limitations and fulfill the growing future demands of sugar, biofuel, and bioenergy industries.

## Abstract (ru)

Гибридизация различных местных сортов или диких видов сельскохозяйственных культур способствует генетической рекомбинации и приводит к созданию улучшенных сортов, в особенности у культур, размножающихся половым путем. В отличие от этого, у культур с бесполым размножением, таких как сахарный тростник (гибрид Saccharum spp.), генетическая рекомбинация посредством гибридизации затруднена из-за само- или перекрестной несовместимости (низкой фертильности). Такие сельскохозяйственные культуры могут быть улучшены за счет сомаклональной изменчивости с помощью методов культуры ткани. Сахарный тростник, являющийся одним из основных источников мирового производства сахара и биоэтанола, из-за недостаточной устойчивости к биотическим и абиотическим стрессовым факторам несет значительные потери урожая. Несмотря на эффективность методов культивирования in vitro, сомаклональная изменчивость остается недостаточно изученной в программах селекции сахарного тростника. Для решения проблем, появляющихся у сахарного тростника в условиях меняющейся динамики окружающей среды, в настоящем обзоре критически оценивается роль сомаклональной изменчивости в создании сортов сахарного тростника, рассмотрены ее основные механизмы, практическое применение и факторы, влияющие на ее возникновение. Кроме того, обсуждаются ограничения и проблемы практического применения этого метода при создании сортов, не позволяющие адекватно использовать его в современных селекционных работах. Использование потенциала сомаклональной изменчивости в сочетании с передовыми технологиями позволит преодолеть данные ограничения и удовлетворить растущие потребности в производстве сахара и биотоплива и в биоэнергетической промышленности.

## Introduction

The plant tissue culture technique is traditionally used for development of uniform genetic plants through micropropagation, which is the main benefit of clonal cultivars used for commercial cultivation (Duta-Cornescu et al., 2023). However, variation in plants generated from any cell or tissue culture under in vitro conditions has been identified and termed as “somaclonal variation” (Larkin, Scowcroft, 1981). This variation provides a potential substitute for crop improvement, especially in asexually propagated crops, such as sugarcane, or crops with narrow genetic bases, self or cross-incompatibility, and irregular inbreeding depression, which are considered significant limitations in using conventional methods for crop improvement. Such genetic improvement assists in reducing the effect of abiotic and biotic stresses in crops like sugarcane via development of resilient cultivars.

**Fig. 1.**
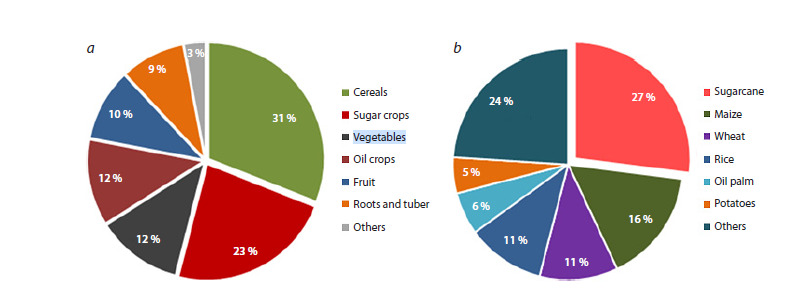
Contribution of sugarcane to global production in 2023 by (a) commodity group and (b) individual commodity. Source: (FAOSTAT, 2024).

Sugarcane is a prominent genetic feedstock used in production of sugar (Azu et al., 2022), biofuel (Marques et al., 2024), and bioenergy (Ahmad, Ming, 2024). Sugar crops rank second in total production among staple crops (Fig. 1a). Among sugar crops, sugarcane yields up to 85 % of global sugar production with the rest contributed by sugarbeet (FAO, 2024). Although a large number of different crops are cultivated worldwide, only four account for half of the global production; sugarcane ranks first, being followed by maize, rice, and wheat, as shown in Figure 1b. Beyond sugar industry, the significance of sugarcane extends from biomass resources to biofuel production (Ahmad, Ming, 2024).Conventional breeding by selecting superior parents for mating is hampered in sugarcane by limitations like asynchronous flowering, low seed viability, low fertility, and lack of flowering caused by environmental conditions (Khan M.T. et al., 2018). In fact, sugarcane flowering is specific to certain regions of the world. Continuous efforts have been made to reduce the generational interval in other crops (e. g: doubled haploid, shuttle breeding, etc.), but sugarcane breeding has not benefited from such techniques

These limitations have led to a narrow genetic base; as the modern genetic feedstock of sugarcane exhibits poor resistance to several diseases (Budeguer et al., 2021). To overcome the common breeding constraints, in vitro culture is of keen interest as a way to generate genetic variability for selecting desired genotypes. Variants with a good yield potential developed through tissue culture in sugarcane can be utilized for facing regional environmental challenges and biotic/abiotic stresses. Sugarcane variants developed from in vitro culture have been reported since 1971 (Heinz, Mee, 1971).

This paper focuses on how somaclonal variation can complement or replace conventional breeding to generate genetic variations among clones in sugarcane. It covers the mechanisms involved in development of variants along with the factors leading to a satisfactory rate of variation. Despite the successful practical application in developing variants in sugarcane, the progress over the last decade has been neglected. Therefore, this review draws attention towards the significance of somaclonal variation by highlighting its limitations, which may constrain its application on a commercial scale.

## In vitro culture techniques

The most promising tissue culture techniques widely adopted in various crops include anther culture, cell suspension culture, callus culture, embryo culture, micropropagation, and protoplast culture. Different researchers have established different protocols for successful induction of somaclonal variation in sugarcane, but detailed discussion of their protocols is beyond the scope of this paper. However, the principles of extensively used techniques for in vitro culture and their regeneration in sugarcane are discussed briefly below.

**Fig. 2.**
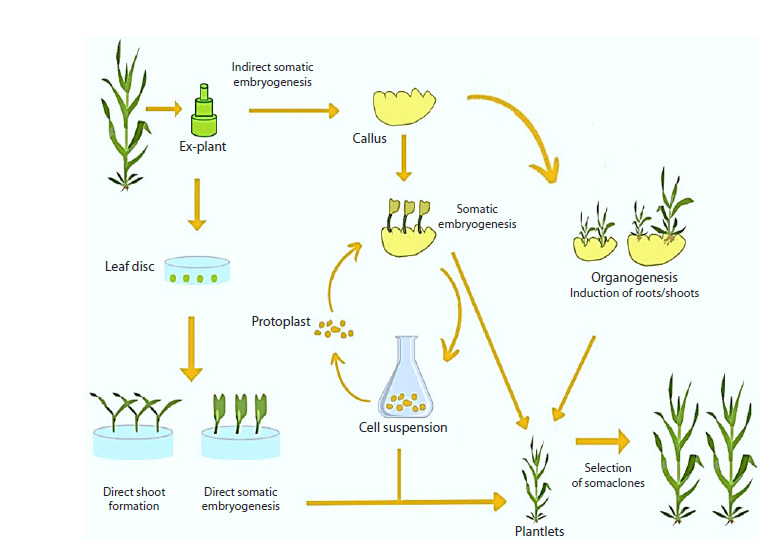
Regeneration pathways in sugarcane

Organogenesis and somatic embryogenesis are the main pathways for in vitro plant regeneration. They require totipotency and competency for plant regeneration under in vitro conditions as shown in Figure 2. Newly regenerated plants need to be acclimated to external growth conditions by placing them under low relative humidity and high illumination, which results in cuticle production (Warren, 1991).

In organogenesis, shoots are induced to differentiate from cells or cell clusters. The process involves the progression of leaves, shoots or roots from the explant and their transfer to a diverse medium for root formation (Bidabadi, Jain, 2020). It can be further classified into direct and indirect organogenesis. In the former, the root or shoot develops directly without going into a callus phase. Thorat et al. (2018) has found that sugarcane plants developed from indirect organogenesis exhibit more genetic variation than those developed from direct organogenesis.

In somatic embryogenesis, a cell or group of somatic cells gives rise to a somatic embryo, which morphologically resembles zygote embryos. The somatic embryo has a bipolar structure giving rise to both shoot and root meristems (Khan I.A., Khatri, 2006). Somatic embryogenesis can also follow the direct or indirect (with an intermediate callus phase) pathways.

The first phase in somatic embryogenesis is the induction of embryogenic cells, which requires time to dedifferentiate. Competence and embryo formation depend upon auxins, mainly in the form of 2,4-dichlorophenoxyacetic acid (2,4-D). It represses morphogenesis and disrupts cell-to-cell interaction, leading to the fragmentation of a single cell or group of cells – the mechanism of the proliferation of embryogenic suspension cultures (De Klerk et al., 1997). It is a commonly used pathway for sugarcane micropropagation (Naz et al., 2008; Almeida et al., 2022), with different established protocols. However, these protocols are efficient for not all genotypes due to their differential behavior (Di Pauli et al., 2021).

Nickell and Maretzki (1969) were first to report cell suspension culture in sugarcane. For this purpose, friable callus is dispersed in a liquid medium and then agitated on a rotary shaker to obtain a culture of isolated cells. This process is genotype-dependent, and those with high phenolic compound levels do not perform well. Generally, suspension cultures have been utilized to study metabolic and physiological processes in sugarcane (Goldner et al., 1991; Thorat et al., 2017; Bottcher et al., 2021). However, Thorat et al. (2017) reported the presence of variation in sugarcane somaclones developed through cell suspension culture, although at a lower frequency, as confirmed by molecular markers.

## Mechanisms of somaclonal variation

Somaclonal variation is a multifaceted phenomenon comprising genetic or epigenetic mechanisms (Leva, Rinaldi, 2017). The stability of mutations of qualitative features in subsequent generations relies on their molecular basis (Hung et al., 2018; Azizi et al., 2020). If these mutations are unstable or not inherited by the next generations, they may be regarded as epigenetic changes (Hung et al., 2018). Moreover, modifications in tails of histones and DNA methylation are also viewed as a part of epigenetic change (Azizi et al., 2020), which are usually caused by residual effects of plant growth regulators in tissue culture media (Jackson, Lyndon, 1990). Chromosomal variations are usually caused by explant source or culture media conditions (Phillips et al., 1994). Such variations also have been observed in sugarcane somaclones (Sobhakumari, 2012).

Somaclonal variation has been observed in several species and extensively utilized due to its commercial potential, irrespective of the type of mechanisms involved (Leva, Rinaldi, 2017). Economically, somaclonal variation should be valuable and heritable. Genetic changes can be induced by a variety of mechanisms, including changes in chromosome structure and number, chromosomal breakage or aberrations (deletions/ inversions), and cryptic changes. Variations in chromosome number, structure, and aberrations are observed in regenerated plants (Mujib et al., 2007). Such changes may lead to loss of genes or their function (Leva et al., 2012). Point mutations responsible for cryptic changes may affect mitochondrial and chloroplast genomes and are typically predicted to take place. Moreover, cryptic changes such as gene silencing or activation of a transposable element play a vital role in somaclonal variation (Barret et al., 2006).

## Factors affecting somaclonal variation

The frequency and type of somaclonal variations in sugarcane can be affected by different factors, such as the genotype composition, explant source, culture medium, time period, and number of culture cycles as discussed below.

**Genotype**

Different plant species or genotypes vary in their susceptibility to somaclonal variation. The most significant factor among various factors may be plant genotype (Shen et al., 2007; Tican et al., 2008; Nwauzoma, Jaja, 2013). Moreover, different genotypes show different responses under stress, pointing out that somaclonal variation is also affected by genotypic composition.

Shen et al. (2007) assessed plant regenerants and showed that plant genotype is a key factor influencing somaclonal variation during in vitro culture. The rate of somaclonal variation varies from 2.6 to 40.4 % depending upon the plant genotypes.

Thus, somaclonal variation is a genotype-dependent phenomenon specifically linked to the regeneration potential of different varieties (Manchanda et al., 2018).

**Explant source**

The origin of the explant exerts an important influence on the rate of somaclonal variation (Ahuja, 1998). Also, it has been reported that the explant source may greatly influence genetic fidelity (Krikorian et al., 1993). Regenerates from explants with pre-existing meristems (such as axillary buds or shoot tips), exhibited less somaclonal variation than those developed from highly differentiated tissue (such as leaves, stems, or roots), which usually produce significant somaclonal variants (Duncan, 1997; Sharma et al., 2007).

However, successful cases of in vitro regeneration using leaf pieces, leaf sheath, shoot apical meristem, or pith have been reported in sugarcane (Ali et al., 2007; Abdullah et al., 2013). A comparative study investigated better sources of explant in sugarcane. Leaves, shoot apical meristem, or pith were used as explant sources. Leaves performed better than all other sources, followed by shoot apical meristem and pith during callus induction (Ali et al., 2007). Moreover, Abdullah et al. (2013) reported the leaf as the explant source that performed best for indirect somatic embryogenesis technique in sugarcane as compared to pith. The genetic fidelity of somaclones developed from leaves as explants showed polymorphism up to 81 %, demonstrating that direct regeneration from immature leaf slices could be helpful in exploitation of genetic variation and improvement of the existing genotype of sugarcane (Khan I.A. et al., 2009).

**Culture medium composition**

From the viewpoint of in vitro culture conditions, the composition of culture media is the most influential element for plant regeneration in sugarcane (Abdullah et al., 2013). For the practical application of in vitro culture, appropriate concentrations and combinations of cytokinins and auxins are necessary (Letham, Gollnow, 1985). The role of cytokinins in the development of shoot apical meristem of sugarcane has been reported in (Ali et al., 2007).

Plant growth regulators in culture media are potent agents of somaclonal variations (Leva et al., 2012). Suboptimal or supraoptimal concentrations of growth regulators, mainly synthetic compounds, have been associated with variation. Somaclonal variation is also favored by rapid, disorganized growth (Karp, 1994). Adding auxin to the culture media of unorganized callus increases the variation by augmenting the rate of DNA methylation (LoSchiavo et al., 1989). Shahid et al. (2012) reported superiority of 2,4-D as an auxin over IAA during callogenesis in sugarcane. Highly significant differences were observed among somaclonal variants developed in sugarcane using different levels of 2,4-D in media, thus pointing out that culture media greatly influence the occurrence of somaclonal variation. In comparison to explant source or plant genotype, remarkable differences were reported for 2,4-D in sugarcane (Abdullah et al., 2013).

2,4-D and kinetin are two common growth regulators that can lead to changes in chromosome number and induce chromosomal abnormalities (Daub, 1986). Growth regulators increase the cell division rate in the culture during the somatic differentiation stage, leading to endopolyploidy, polyteny, and chromosomal changes (D’Amato et al., 1977; Larkin, Scowcroft, 1981). Culture medium conditions are often responsible for chromosomal variations (Phillips et al., 1994). Such variations have also been observed in sugarcane somaclones (Sobhakumari, 2012). Khan I.A. et al. (2008) also reported 2,4-D as the best source of somaclonal variation when comparing different auxins (Dicamba, Picloram, and IAA) in sugarcane. The maximum number of chlorophyll mutants was developed with 2,4-D as compared to other auxins, representing the highest rate of somaclonal variation in sugarcane. Plants obtained with 2,4-D auxin showed higher phenotypic variability in sugarcane mainly due to the true change in genetic makeup while showing its enhancing effect on cane yield and plant height.

**Culture age and number of subcultures**

Somaclonal variation increases with the duration of the culture (Farahani et al., 2011; Sun et al., 2013). As callus age increases, chances of diverse plant production increase during successive subcultures (Zayova et al., 2010). Khan S. et al. (2011) showed that somaclonal variation increased after the eighth subculture.

However, not only the number of subcultures but also the duration of each subculture is significant in creating somaclonal variation particularly in callus culture and cell suspension (Bairu et al., 2006; Sun et al., 2013). Somaclonal variation is more promising in plants regenerated after longer culturing, as rapid multiplication of tissue may influence the genetic stability (Israeli et al., 1995; Etienne, Bertrand, 2003; Petolino et al., 2003). About 30 % of genetic variations marked by morphological changes appeared in sugarcane clones after the fourth subculture (Nogueira et al., 2022).

## Detection of somaclonal variation

Detection of somaclonal variation is the foremost step in selecting a cultivar with desirable traits or discarding material with unwanted characteristics. Various biochemical, cytological, morphological, and molecular approaches have been employed to detect the extent and type of somaclonal variation in sugarcane.

Somaclonal variants can be simply assessed by morphological traits such as abnormal pigmentation, plant height, or leaf morphology (Israeli et al., 1991).

The existence of somaclonal variation among in vitro-cultured sugarcane plants has been shown by different researchers using morphological markers (Doule, 2006; Dalvi et al., 2012; Khan I.A. et al., 2015; Yasmeen et al., 2017; Abo-Elwafa et al., 2021). In sugarcane variety CoJ 64, morphological variations in traits such as leaf length, stalk diameter, etc. were observed during three years (Sobhakumari, 2012). Likewise, Rajeswari et al. (2009) reported 51 commercially promising sugarcane somaclones out of 700 and evaluated their genetic variability using morphological traits. Morphological markers have been widely used in assessment of genetic variations among sugarcane somaclones (Khan I.A. et al., 2015; Yasmeen et al., 2017; Abo-Elwafa et al., 2021). Nonetheless, the detection of somaclonal variants using morphological tools is environmentally sensitive and time-consuming (Bairu et al., 2011).

Change in chromosome number or structure is assessed by cytological analysis (Sobhakumari, 2012; Abreu et al., 2014). Flow cytometry and complex microscopic techniques are usually used to detect somaclonal variation in tissue-cultured plants. Flow cytometry is widely accepted (Doležel et al., 2004), though it is time-consuming and chemicals used to prepare cytosolic suspensions may interfere with DNA content (Krishna et al., 2016).

In characterization of the complex genome of sugarcane, genetic difference has been verified by flow cytometry (Oliveira et al., 2015; Metcalfe et al., 2019). Thus, Oliveira et al. (2015) detected variation in DNA content in sugarcane varieties and proposed a reliable analysis technique for flow cytometry. Sixteen varieties were classified into four groups relative to their DNA content. Similarly, Nogueira et al. (2022) observed changes in DNA content in regenerated sugarcane plants. Three out of ten genotypes showed change in DNA content. Among these three, two showed reduction of 0.45 pg DNA, while one genotype showed an increase up to 61 % in DNA content during in vitro culture. This increase in genome size might be due to polyploidy or transposable elements (Bennetzen et al., 2005), whereas the decrease might be caused by chromosomal breakage or deletions (Petrov, 2001).

Phenotypic variations among organisms are the outcome of biochemical variations that can be due to polymorphism in the genetic makeup of organisms. Isozyme analysis was first performed in sugarcane in 1969 (Heinz, 1969). Enzymes, such as peroxidase, superoxide dismutase, and malate dehydrogenase were used to study variation in sugarcane (Weising et al., 2005). Sugarcane somaclones showed improved activity of antioxidants like catalase, superoxide dismutase, peroxidase, ascorbate peroxidase, and ascorbate under drought stress (Naheed et al., 2020). Variation in sugarcane based on biochemical parameters was also reported by Yasmeen et al. (2017) and Dalvi et al. (2012). Nevertheless, the implementation of isozyme analysis is limited due to some limitations, such as their being tissue-specific, few in numbers, and being affected by environmental conditions.

The complete picture of variations in complex plant genomes can be assessed with the help of molecular markers. They are more reliable tools, as compared to biochemical markers, as they are more numerous, environment-independent, reproducible, and highly specific (Manchanda et al., 2018). Currently, different molecular markers, such as amplified fragment length polymorphism (AFLP), randomly amplified polymorphic DNA (RAPD), simple sequence repeats (SSR), and inter-simple sequence repeats (ISSR), are utilized in different plants to study somaclonal variation (Leva et al., 2012). Somaclonal variation was widely detected in sugarcane by using molecular markers (Thumjamras et al., 2011; Seema et al., 2014; Mahmud et al., 2015; Tawar et al., 2016). In particular, SSR markers are frequently utilized to study variation among somaclones in sugarcane (Nair et al., 2002). Smiullah et al. (2012) rated SSR markers the best tool to study genetic diversity and found up to 51 % polymorphisms using SSR primers in sugarcane somaclones.

## Achievements through somaclonal variation in sugarcane

The success of any conventional breeding program depends on genetic variability. However, a program lasts for about 10–15 years and comprises genotype selection and variety testing, passing through different crop development stages. Therefore, to boost the variation, especially in asexually propagated crops, plant tissue culture presents a novel technique, which assists breeders in creating variability in crops in a short time (Karp, 1992; Mathur, 2013). Tissue culture techniques significantly contributed to crop improvement in sugarcane. Useful somaclones have been developed around the globe against different biotic and abiotic stresses (Oloriz et al., 2011; Dalvi et al., 2012; Kumar et al., 2012; Nikam et al., 2015).

The most significant somaclones developed mainly during the last two decades, along with their improved features, are shown in the Table. Ono was one of the pioneer tissue-cultured sugarcane varieties developed from the susceptible variety Pindar that was found resistant to Fiji disease (Krishnamurthi, Tlaskal, 1974). Two somaclonal variants of sugarcane variety CoC 671 were released as cultivars: Phule Savitri, which has high sucrose content, early maturity, and moderate tolerance to smut and red rot (Jalaja et al., 2006), and VSI 434, which showed an almost double increase in sugar and cane yield (Tawar et al., 2016). After VSI 434, no significant commercially released variety or clones have been produced in the last decade, as shown in the Table.

**Table 1.**
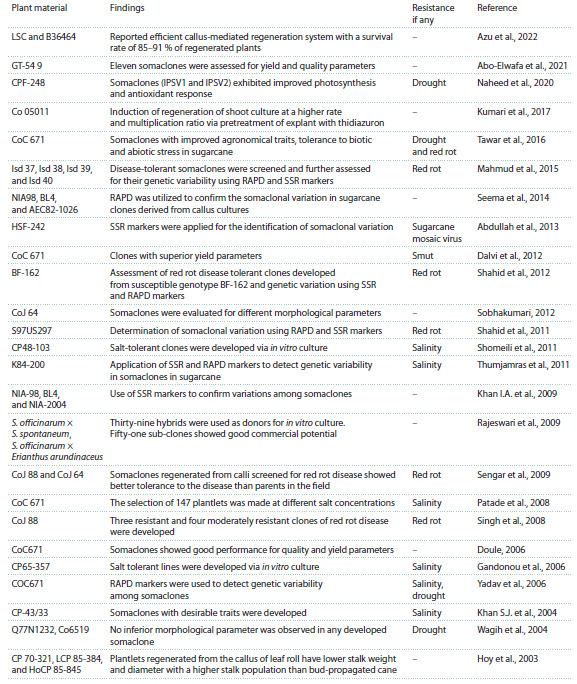
Promising clones developed in sugarcane

## Challenges encountered in somaclonal variation

Regardless of various somaclones developed in sugarcane, genetic improvement remains limited with few varieties released to date. Some distressing concerns hindering its practical application on commercial scale are listed below:

• Quantitative inheritance in sugarcane is a major limiting factor for the generation of favorable mutations via tissue culture, explicitly regarding yield and sucrose content. • Somaclonal variation is an expensive technique, as it demands a closed facility. • The rate of somaclonal variation depends on the genotype of explant used. • Somaclones exhibit uncontrolled and unpredictable variation. • Most of the traits acquired by this variation are uncontrolled, unstable, and epigenetic in nature. Therefore it is essential to study the genetic diversity of tissue-cultured plants after their transplantation into field. • Multilocation trials are demanded to test the stability of desired traits over generations. • To assess the genetic stability of somaclones, extensive field trials are required. • Issues such as poor acclimatization, contamination, and tissue dying also impede the practical application of variant development.

To overcome these limitations, somaclones can serve as an initial material for plant breeding rather than as finished cultivars. They act as donors of specific valuable traits, requiring further improvement through conventional breeding methods. Integrating conventional breeding with tissue culture technique, along with other advanced molecular tools such as marker assisted selection, genetic mapping, or gene editing tool (e. g. CRISPR-Cas9) or mutation can enhance sugarcane resilience to abiotic and biotic stresses. Moreover, gene editing approaches enable the modification of targeted genes linked to stress tolerance, ultimately improving the resistance mechanism in sugarcane. Implementing these advance approaches can help breeders overcome the aforesaid concerns.

## Conclusions and future prospects

Genetic variability exists both in wild-type and commercial varieties, serving as the foundation for improving cultivars. However, in sugarcane, aspects such as the complex genome, narrow genetic pool, long breeding cycle, and low fertility are the key limiting factors for the development of superior cultivars via recombination. In this context, the tissue culture technique can overcome the hardship of creating genetic variation in sugarcane. Somaclonal variation induced by tissue culture has become the most prevalent approach in sugarcane for developing varieties and superior lines or clones with tolerance to biotic or abiotic stresses, particularly in regions where climatic conditions limit fertilization.

Despite of its potential, somaclonal variation has been underexplored in recent years due to several challenges. These challenges can be met by integrating the tissue culture technique with other biotechnological or latest approaches. For instance, identification of variants at an early stage by means of molecular markers can be productive, as early selection or elimination of variants reduces the time expenditure, especially in long duration crops like sugarcane. Combinatorial use of induced mutagenesis with in vitro culture can further enhance variation frequency in crops with large complex genomes and lead to development of sugarcane for commercial use under changing environmental conditions. Moreover, harnessing genetic edition tools like CRISPR-Cas9 can enhance crucial traits related to biofuel and bioenergy production and fulfill the growing future demands in sugar, biofuel, and bioenergy industries.

## Conflict of interest

The authors declare no conflict of interest.
